# Interaction between the Haptoglobin 2 Phenotype and Diabetes Mellitus on Systolic Pulmonary Arterial Pressure and Nitric Oxide Bioavailability in Hemodialysis Patients

**DOI:** 10.1155/2015/613860

**Published:** 2015-06-11

**Authors:** Inbal Dahan, Evgeny Farber, Nadia Thauho, Nakhoul Nakhoul, Adi Francis, Mohamad Awawde, Andrew P. Levy, Daniel B. Kim-Shapiro, Swati Basu, Farid Nakhoul

**Affiliations:** ^1^Diabetic Nephropathy Laboratory, The Baruch Padeh Poriya Medical Center, Faculty of Medicine in the Galilee, 15208 The Lower Galilee, Israel; ^2^Division of Nephrology & Hypertension, The Baruch Padeh Poriya Medical Center, Faculty of Medicine in the Galilee, 15208 The Lower Galilee, Israel; ^3^Division of Vascular Medicine, Holy Family Hospital, 16234 Nazareth, Israel; ^4^Rappaport Faculty of Medicine, Technion-Israel Institute of Technology, 31096 Haifa, Israel; ^5^Department of Physics and Translational Science Center, Wake Forest University, Reynolda Campus, Winston-Salem, NC 27109, USA

## Abstract

Elevated systolic pulmonary artery pressure (s-PAP, ≥35 mmHg) serves as an independent predictor of mortality in hemodialysis (HD) and diabetic (DM) patients. A polymorphism in the antioxidant Haptoglobin (Hp) gene has been shown to regulate the bioavailability of nitric oxide (NO), a major mediator of pulmonary vascular tone. We therefore set out to test the hypothesis that the Hp polymorphism may be a determinant of developing elevated s-PAP specifically in the DM state due to a decreased bioavailability of NO. To test our hypothesis we Hp typed and performed transthoracic echocardiography on a series of HD patients and stratified them into elevated and normal s-PAP groups and then evaluated whether there was a significant association between the Hp type, elevated s-PAP, and decreased NO bioavailability as defined by low plasma nitrite. We found a statistically significant interaction between the Hp type and DM on the prevalence of elevated s-PAP and lower mean nitrite levels with the combination of elevated s-PAP and low nitrite levels being significantly more prevalent in Hp 2-2 DM individuals. We conclude that the Hp 2 type is associated with elevated s-PAP levels and low plasma nitrite levels in HD patients specifically in the DM state.

## 1. Introduction

Pulmonary hypertension (PHT) is an elevation of systolic pulmonary arterial pressure (s-PAP) and resistance that can be the result of heart, lung, or systemic disorders [1, 2]. It is defined as a mean s-PAP exceeding 30 mmHg as measured by right heart catheterization (invasive gold standard) or as an estimated pulmonary artery pressure (e-PASP) exceeding 35 mm/Hg level as measured by Doppler echocardiography [1–3].

PHT involves vasoconstriction and obliteration of the lumen of small vessels in the lungs by plexiform lesions resulting in increased resistance to flow [1–3]. Recently, high e-PASP was recognized as a complication of chronic kidney disease (CKD) and end-stage renal disease (ESRD) [1, 2, 4–6]. We have previously shown a 40% incidence of unexplained high e-PASP in patients with ESRD on chronic hemodialysis (HD) therapy [2, 7–12] and that high e-PASP serves as an independent predictor of increased morbidity and mortality in HD patients [1, 2, 4, 8, 13–15]. Therefore, understanding the factors that contribute to high e-PASP in HD patients is of critical importance. Normally, pulmonary endothelial cells increase the synthesis of nitric oxide (NO) in response to increased pulmonary blood flow and pressure in an attempt to restore normal vascular tone [8]. Therefore, factors which may decrease NO bioavailability may directly impact e-PASP in HD patients.

Hp is an acute phase protein that acts as an antioxidant by virtue of its ability to bind free hemoglobin (Hb) and prevent heme-iron mediated oxidation [16–18]. When Hb is released into the circulation, it binds immediately to Hp to form a Hp-Hb complex that is rapidly removed predominately by the macrophage CD163 scavenger receptor expressed on Kupfer cells in the liver [18–23]. A polymorphism at the Hp locus (rs72294371) is associated with three possible Hp genotypes and protein phenotypes denoted 1-1, 2-1, and 2-2 [24]. The Hp protein products of the Hp 1 and Hp 2 alleles differ in both their biochemical and their functional properties such as the ability to serve as an antioxidant and to clear Hb from the plasmatic compartment [19, 21, 25] and these differences are exaggerated in Diabetes Mellitus (DM). The importance of the aforementioned differences in Hb clearance by Hp type is that extra corpuscular Hb has been shown to markedly reduce NO bioavailability in serum [26, 27]. In this study, we set out to test the hypothesis that individuals with the Hp 2 allele would have higher e-PASP specifically in the DM state due to a decreased bioavailability of NO.

## 2. Subjects and Methods

### 2.1. Study Design

The current study is a multicenter cross-sectional analysis that was carried out to assess whether the Hp 2 phenotype (Hp 2-2 or Hp 2-1) is associated with elevated e-PASP and decreasing NO bioavailability in DM and non-DM HD patients. The study included 122 HD patients from Baruch Padeh Medical Center and the Holy Family Hospital. 80% of these patients, without differences between the different Hp types, are under chronic treatment with Beta-Blockers, Calcium Channel Blockers, and Angiotensin Receptor Blockers (ARB) for arterial hypertension. Less than 10% of these patients are treated with statins for hypercholesterolemia, due to the high incidence of side effects in HD. All experiments were approved by the Helsinki Committee of both hospitals and informed consent was obtained from all participants. The study participants' characteristics are presented in Table 1. During data analysis, we stratified the patients into two groups: patients with high e-PASP defined by an e-PASP ≥ 35 mmHg at rest and patients with normal e-PASP defined by e-PASP < 35 mmHg, and we then evaluated whether there was a significant association between Hp 2 phenotype and elevated e-PASP levels and between the Hp 2 phenotype and decreased NO bioavailability as defined by low plasma nitrite levels (less than 0.2 *μ*M). Furthermore to evaluate the influence of DM on the frequency of high e-PASP or on the frequency of low plasma nitrite levels, we stratified members of each group (high and normal e-PASP) into those with and without DM.

### 2.2. Subjects

The study includes 122 HD patients (males and females), 81 patients with DM and 41 HD patients without DM. Exclusion criteria were severe lung disease, congestive heart failure, pregnancy, and any malignancy. Demographic and clinical data concerning patients (age, sex, diabetic status, comorbidities, and medications used) were obtained directly from the patients or from their hospital files.

### 2.3. Haptoglobin Typing

Hp phenotyping was performed on plasma samples by polyacrylamide gel electrophoresis as previously described [28]. In brief, 10 *μ*L of Hb-enriched plasma was subjected to electrophoresis in a nondenaturing gel, and the gel was subsequently immersed in solution containing a congener of benzidine with a precipitate forming in the gel corresponding to the location of Hb-Hp complexes. The Hp type of the sample was determined by the banding pattern of the Hp-Hb complexes with each of the three Hp phenotypes having a characteristic banding fingerprint.

### 2.4. Echocardiographic Measurements of PASP

Estimated systolic pulmonary pressure (e-PASP) was estimated by Doppler echocardiography as described by Gladwin et al. [15]. To avoid overestimation of e-PASP due to volume overload, echo studies in the HD patients were performed within 1 h after completion of HD, when the patients were at optimal dry weight according to hydration status, blood pressure, and weight. Two experienced operators performed all the echocardiographic studies, using an Acuson Sequoia, Aspen, or 128 XP (Mountain View, CA) ultrasound machine. A complete two-dimensional M-mode and Doppler echocardiographic study was obtained from each patient. A tricuspid regurgitation systolic jet was recorded from the parasternal or apical window with the continuous-wave Doppler probe. Systolic right ventricular (or pulmonary artery) pressure was calculated using the modified Bernoulli equation: PAP = 4 × (tricuspid systolic jet)^2^ + 10 mmHg (estimated right atrial pressure). High e-PASP was defined as an e-PASP ≥ 35 mmHg at rest.

### 2.5. Measurement of Plasma Nitrite

Plasma nitrite was used as a measure of NO bioavailability as this measure has been shown to reflect NOS function [29–32]. For this measurement, blood was drawn into Lithium-Heparin tubes and immediately centrifuged for 2 minutes and the plasma was quickly frozen in dry ice and stored at −20°C until the nitrite measurements were performed. Plasma was mixed with an equal volume of 100% methanol, vortexed, and centrifuged at 11,500 g for 10 minutes. The supernatant was loaded into 96-well plates and nitrite concentrations were measured using an ENO-20 NO_*x*_ analyzer (EICOM, San Diego, CA, USA). The nitrite was separated via column chromatography and reacted individually with the Griess reagent to synthesize a red diazo compound that was read at a wavelength of 540 nm by a visible light detector in the ENO-20. A level of plasma nitrite of below 0.2 *μ*M was defined as low based on previous studies [33–40] demonstrating that the normal mean plasma nitrite is approximately 0.2 *μ*M.

### 2.6. Statistics

Patients were stratified into high e-PASP (≥35 mmHg) and normal e-PASP groups. Cross-tabulation and Pearson's chi-square tests were used to evaluate the prevalence of the three different Hp phenotypes in each group. Logistic regression analysis was used to evaluate interaction between Hp phenotypes and DM on high e-PASP levels. In this logistic regression model, the e-PASP group (high versus normal) was set as a dependent variable (binary outcome). Hp phenotypes, DM, and the interaction of Hp phenotype and DM were set as independent variables (covariates). Logistic regression analysis was also used to evaluate interaction between Hp phenotypes and DM on low levels of nitrite (<0.2 *μ*M). In this logistic regression model, the nitrite levels group (above 0.2 *μ*M versus less than 0.2 *μ*M) was set as a dependent variable (binary outcome). Hp phenotypes, DM, and the interaction of Hp phenotype and DM were set as independent variables (covariates). Proportion test, *t*-test, chi-square test, and Fisher's exact tests were used to determine significant associations between Hp phenotype and high e-PASP as well as between Hp phenotype and plasma nitrite levels. ANOVA tests were used to define significant differences in nitrite mean levels according to the Hp phenotype and between groups. Data analysis was done using IBM SPSS software version 20.0. The data are shown as mean ± SEM and a probability *P* < 0.05 was considered statistically significant.

## 3. Results

### 3.1. E-PASP Is DM and Hp Type Dependent

**Figure 1:**
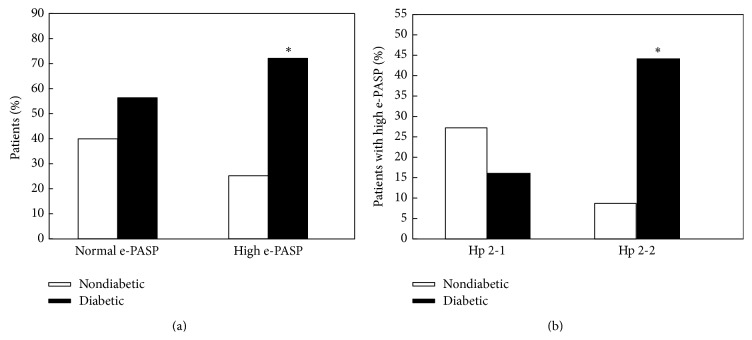
E-PASP is increased in DM and in Hp 2-2. (a) Association of e-PASP and DM. Among individuals with high e-PASP, the prevalence of DM was significantly increased (^*∗*^ *P* = 0.0001 compared to non-DM individuals). There was no relationship between DM and e-PASP in individuals with normal e-PASP. (b) Association of Hp phenotype and e-PASP. DM dependent significant differences in the prevalence of high e-PASP seen in (a) above were only in study participants with the Hp 2-2 phenotype (^*∗*^ *P* = 0.0001 comparing prevalence of high e-PASP in Hp 2-2 participants with and without DM).

PHT was defined as an estimated systolic pulmonary pressure (e-PASP) exceeding 35 mmHg by Doppler echocardiography. As shown in Figure 1(a) among individuals with high e-PASP there was a significant increase in DM as compared to non-DM individuals (*P* = 0.0001). Furthermore, as demonstrated in Table 2, the prevalence of high e-PASP was significantly increased (*P* = 0.018) only in individuals with the Hp 2 phenotype (Hp 2-2 and Hp 2-1). Logistic regression analysis demonstrated a statistically significant interaction between the Hp phenotype and DM on the prevalence of elevated e-PASP (*P* = 0.018) with elevated e-PASP being significantly more prevalent in Hp 2-2 DM individuals (*P* = 0.0001) (Figure 1(b)).

### 3.2. DM and Hp-Dependent Differences in Mean Plasma Nitrite Levels of HD Patients

**Figure 2:**
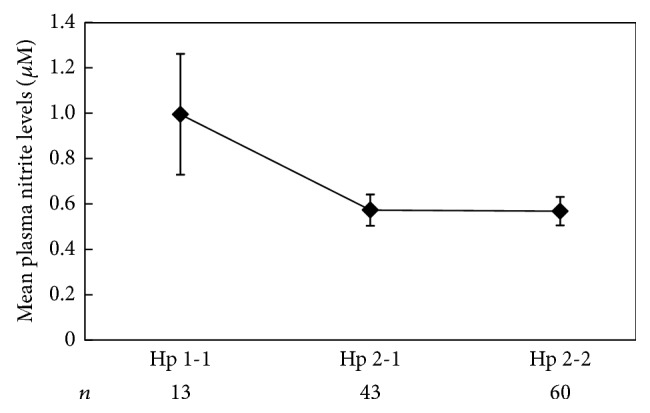
Lower mean nitrite levels in Hp 2 participants. Mean nitrite levels were significantly higher in Hp 1-1 compared to Hp 2 participants (*P* = 0.034).

Endothelial dysfunction contributes to increased rates of high e-PASP in DM and in HD patients. Hence, we sought to determine whether there were DM and Hp-dependent differences in NO bioavailability by measuring plasma nitrite levels of HD patients [32]. When comparing nitrite mean levels across the three Hp phenotypes, we found significantly lower mean nitrite levels in study participants with the Hp 2 phenotype (Hp 2-2 or Hp 2-1) compared with participants with Hp 1-1 phenotype (Figure 2). The Hp-dependent differences in the mean nitrite levels were statistically significant among DM (*P* = 0.043) but not in non-DM study participants (*P* = 0.466).

### 3.3. Hp Genotype Dependent Differences in the Prevalence of NO Bioavailability

**Figure 3:**
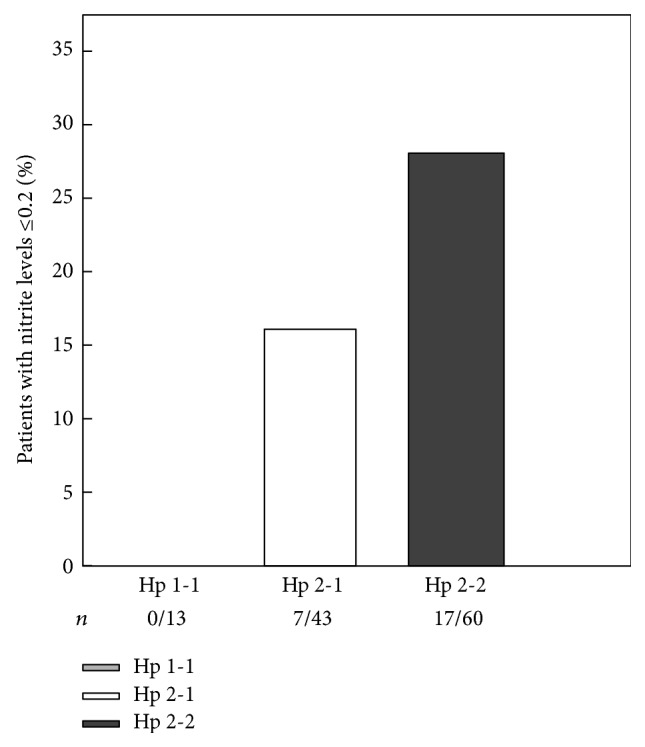
Hp phenotype dependent differences in the prevalence of low plasma nitrite. None of the individuals with Hp 1-1 phenotype had nitrite levels below 0.2 *μ*M compared with 16.3% of the Hp 2-1 or 28.3% of the Hp 2-2 (*P* = 0.049).

Low NO bioavailability as defined in the methods was a plasma nitrite of less than 0.2 *μ*M. Logistic regression analysis demonstrated a significant effect of Hp phenotype on the prevalence of low NO bioavailability (*P* = 0.028). As shown in Figure 3, none of the Hp 1-1 study participants had nitrite levels below 0.2 *μ*M compared with 16.3% of the Hp 2-1 and 28.3% of the Hp 2-2 phenotype participants (*P* = 0.049).

### 3.4. Low Nitrite Levels and DM Correlate with Elevated e-PASP Levels

**Figure 4:**
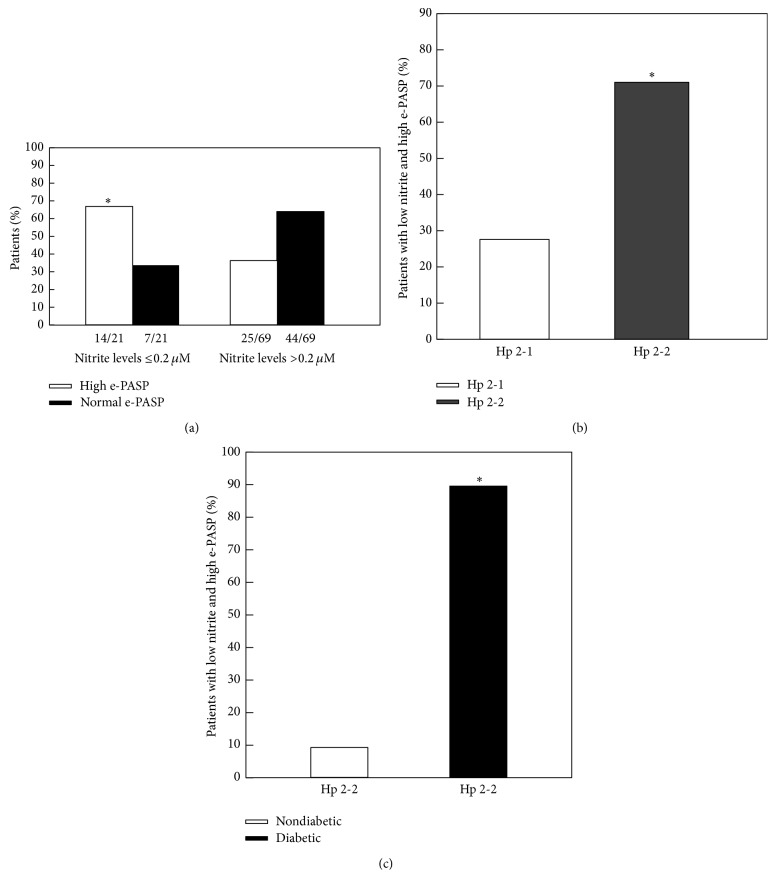
Decreased NO bioavailability is associated with e-PASP, DM, and the Hp 2-2 phenotype. (a) Association of low plasma nitrite and e-PASP. 67% of study participants with nitrite levels below 0.2 *μ*M had high e-PASP levels while only 36% of individuals with normal nitrite levels had high e-PASP (^*∗*^ *P* = 0.003). (b) Association of the Hp phenotype and low plasma nitrite and high e-PASP. 71% of individuals with low nitrite levels and high e-PASP had the Hp 2-2 phenotype (^*∗*^ *P* = 0.023 compared to Hp 2-1 phenotype). (c) Association of DM and the Hp 2-2 genotype with low plasma nitrite and high e-PASP. 90% of all Hp 2-2 individuals who had low nitrite and high e-PASP had DM (^*∗*^ *P* = 0.0003 comparing prevalence of DM versus non-DM in this Hp 2-2 cohort with low nitrite and high e-PASP).

Logistic regression analysis demonstrated a statistically significant relationship between low nitrite levels (below 0.2 *μ*M) and the prevalence of high e-PASP (*P* = 0.017). As shown in Figure 4(a), 67% of study participants with nitrite levels below 0.2 *μ*M had high e-PASP levels while only 36.2% of study participants with normal nitrite levels had high e-PASP (*P* = 0.003). Among those individuals with low nitrite levels and high e-PASP, 71% were Hp 2-2 (*P* = 0.023 compared with Hp 2-1 genotype, Figure 4(b)) and 90% of these Hp 2-2 individuals had DM (Figure 4(c)).

## 4. Discussion

Elevated e-PASP is an independent risk factor for increased morbidity and mortality in dialysis patients. In this study, we have demonstrated that there is a highly significant interaction between the Hp phenotype and DM on the prevalence of high e-PASP in individuals undergoing dialysis with a marked increase in the prevalence of high e-PASP in individuals with the Hp 2-2 phenotype and DM. These data also demonstrate that the Hp 1-1 phenotype may exert a protective role in dialysis patients against developing high e-PASP.

The increased risk of developing high e-PASP in Hp 2-2 DM was associated with a marked reduction in NO bioavailability in this study. These results are consistent with recently published work demonstrating that the Hp polymorphism may modulate NO bioavailability in preeclampsia [25].

NO is known to play a key role in regulating pulmonary vascular tone. Free extra corpuscular Hb is increased in other conditions with pulmonary hypertension, including pulmonary embolism, and this condition is associated with increased NO consumption [41].

Accordingly, differences between individuals in factors that influence the clearance of Hb may influence NO bioavailability and increasing the steady state concentration of free Hb could cause pulmonary hypertension. We have previously demonstrated an interaction between the Hp genotype and DM on the clearance of free Hb [42] and on the expression of CD163, the Hp-Hb scavenger receptor in man [18]. We hypothesize that the major reason for the reduced bioavailability of NO in the plasma Hp 2-2 DM individuals is due to increased plasma Hb in these individuals due to a slower clearance of Hb in this population. In Hp 2-2 DM individuals, we have shown that this Hb is tethered to HDL and Hb tethered to HDL has been shown to reduce NO bioavailability [43, 44]. The tethering of Hb to HDL is via a specific interaction of Hp with helix 6 of ApoA1, the major protein component of HDL. Every day approximately 650 mg of Hb is released into the blood stream and the fate of this extra corpuscular Hb is Hp genotype dependent with the Hp-Hb complex being cleared from the plasmatic compartment by the macrophage CD163 receptor. The Hp 1-1 protein clears Hb more quickly than the Hp 2-2 protein and so there is more Hp 2-2-Hb available to bind to ApoA1 in Hp 2-2 individuals [45, 46]. NO bioavailability may also be reduced to increased presence of reactive oxygen species (ROS) that react with NO and that are formed due to redox activity of cell-free hemoglobin [43, 44] which is increased in Hp 2-2 DM individuals. While it is known that drugs can also influence plasma nitrite levels (in particular statins) [47], we believe that this is unlikely to explain the current findings as less than 10% of these patients are treated with statins with no Hp or DM dependent differences in drugs administered in this study cohort.

## 5. Conclusion

In conclusion, these studies suggest a potential mechanism explaining why Hp 2-2 DM may predispose to high e-PASP and possibly a clinically relevant target endpoint for interventional studies to try to reduce morbidity and mortality in Hp 2-2 DM dialysis patients. If these data are confirmed, patients entering the HD program might be screened for Hp phenotype and e-PASP and for patients with preexisting elevated e-PASP peritoneal dialysis or preemptive kidney transplantation could be considered. These studies suggest that extra corpuscular Hb derived iron may play a major role in developing elevated e-PASP and therefore strategies to reduce NO scavenging by this extra corpuscular Hb iron via iron chelation therapy may reduce e-PASP. It is of considerable interest that a recent study [48] showing marked benefit from chelation therapy on cardiovascular disease in DM may have been mediated through reducing iron mediated vascular disease in Hp 2-2 DM individuals. It is therefore sobering that perhaps the high doses of IV iron which the HD population is currently receiving may have untoward effects.

## Figures and Tables

**Table 1:** Study participants' characteristics stratified by Hp phenotype.

	Hp 1-1	Hp 2-1	Hp 2-2
	*n* = 13	*n* = 45	*n* = 64
DM/non-DM	9/4	30/15	42/22
% DM	69	67	66

Mean age (years)	66.1 ± 16.6	68.3 ± 11.0	66.3 ± 14.0
Range	34–82	46–86	36–92

Male/female	9/4	26/19	36/28
M/F ratio	2.25	1.4	1.3

Systemic hypertension % (*n*)	85 (11)	87 (39)	84 (54)

Mean systemic blood pressure	144/74 ± 22/16	148/74 ± 23/13	141/73 ± 29/16

Duration of dialysis (years)	6.5 ± 5.3	4.34 ± 2.12	4.27 ± 2.73
Range	1–15	1–10	1–13

**Table 2:** Frequency of the different Hp phenotypes in normal and high e-PASP groups.

Hp phenotype	Normal e-PASP (e-PASP <35 mmHg) % (*n*)	High e-PASP (e-PASP ≥35 mmHg) % (*n*)	*P* value
Hp 1-1	16.7 (11)	3.6 (2)	0.019^*∗*^
Hp 2-1	31.8 (21)	42.9 (24)	0.208
Hp 2-2	51.5 (34)	53.6 (30)	0.818
Total	100 (66)	100 (56)	

^*∗*^ *P* = 0.019 comparing the prevalence of Hp 1-1 phenotype in normal e-PASP group versus the high e-PASP group.
